# Intensive care unit length of stay and mortality comparison between on-pump and off-pump coronary artery bypass graft: a retrospective study

**DOI:** 10.1186/s43044-023-00374-1

**Published:** 2023-06-12

**Authors:** Rita Zahara Ibrahim, Ericko Ongko Joyo

**Affiliations:** Department of Cardiology and Vascular Medicine, National Cardiovascular Center Harapan Kita, Letjen S. Parman Kav 87 Slipi, West Jakarta, Jakarta, 11420 Indonesia

**Keywords:** Coronary artery bypass, Off-pump, Length of stay, Mortality

## Abstract

**Background:**

Recently, coronary artery bypass graft** (**CABG) techniques, both on-pump (ONCABG) and off-pump (OPCABG), were compared to seek the most effective approach to reduce the cost of prolonged intensive care unit length of stay (ICU LOS) and mortality. This study aims to compare ICU LOS and mortality in ONCABG and OPCABG.

**Results:**

Demographic data of 1569 patients show the variance of characteristics. The analysis shows significant and longer ICU LOS in OPCABG than ONCABG (2.151 ± 0.100 vs. 1.573 ± 0.246 days; *p* = 0.028). Similar results were demonstrated after adjustment of covariates effects (3.146 ± 0.281 vs. 2.548 ± 0.245 days; *p* = 0,022). Logistic regression shows no significant difference in mortality in OPCABG and ONCABG, both in the unadjusted (OR [CI 95%] 1.133 [0.485–2.800]; *p* = 0.733) and the adjusted models (OR [CI 95%] 1.133 [0.482–2.817]; *p* = 0,735).

**Conclusion:**

ICU LOS was significantly longer in OPCABG patients than in ONCABG patients in the author's centre. There was no significant difference in mortality between the two groups. This finding highlights a discrepancy between recently published theories and the practices observed in the author's centre.

## Background

Cardiovascular disease (CVD) has become the leading cause of death globally in developed and developing regions. Among various CVDs, coronary artery disease (CAD) has emerged as the most prevalent subtype, contributing significantly to the burden of CVD-related morbidity and mortality. To manage advanced CAD, coronary artery bypass graft (CABG) surgery has been widely adopted as a crucial therapeutic option aimed at improving survival rates and enhancing the quality of life for affected individuals.

Traditional CABG surgery involves cardiopulmonary bypass (on-pump CABG), temporarily stopping the heart and allowing surgeons to perform grafting procedures. At the same time, blood circulation is maintained through a heart–lung machine. However, in the early 1990s, an alternative technique called off-pump CABG (OPCABG) was introduced to reduce potential complications associated with on-pump CABG, such as systemic inflammatory response syndrome and neurocognitive deficits [1]. OPCABG allows surgeons to perform bypass grafting while the heart is still beating, thereby avoiding cardiopulmonary bypass and its associated risks.

The choice between on-pump and off-pump CABG remains controversial among cardiovascular surgeons and researchers. Numerous studies have investigated the outcomes and advantages of both techniques, leading to conflicting results. Some studies have reported comparable long-term survival rates, intensive care unit (ICU) length of stay, and perioperative mortality between on-pump and off-pump CABG [2, 3]. On the other hand, other studies have suggested potential benefits of one technique over the other regarding reduced complications and improved patient outcomes [4, 5].

In light of the existing controversies surrounding on-pump and off-pump CABG, our study aims to investigate and compare ICU length of stay and mortality outcomes between these two techniques in the Indonesian patient population. Our study distinguishes itself from previous publications by examining a specific patient population and providing contradictory findings to the commonly reported outcomes. Our results have the potential to significantly impact clinical decision-making by challenging the prevailing perceptions and guiding surgeons towards a more individualized approach in the management of advanced CAD.

## Methods

This is a retrospective observational study. Secondary data were collected from medical records of post-CABG patients from June 2010 until January 2014. Ethics approval and waiver of consent were granted by the Ethical Committee Board of National Cardiovascular Center Harapan Kita Hospital No.0449/UN2.F1/ETIK/2016. Consent was waivered because this study only used secondary data, which involved no risk to the subject.

Patients with a confirmed diagnosis of advanced CAD who underwent either on-pump or off-pump CABG surgery were included in the study. We excluded patients with the following condition: (1) cardiogenic shock, (2) on intra-aortic balloon pumps, (3) severe comorbidities that could significantly impact outcomes (malignancies, end-stage renal disease, advanced liver disease), and (4) incomplete medical records.

Descriptive statistics were reported for all variables of interest. Mean and standard deviation were reported for continuous outcomes. Frequency and percentages were reported for categorical outcomes. Logistic regression models were used to determine the death probability between on-pump coronary artery bypass graft (ONCABG) and off-pump coronary artery bypass graft (OPCABG) patients. The odds ratios were computed using the ONCABG group as the reference. Generalized linear models were fitted assuming gamma-distributed errors and a natural log link function to compare intensive care unit length of stay (ICU LOS) between the two groups. The distribution was chosen due to the skewness of the data. All models were fitted in bivariate and multivariate forms to account for the multiple factors that might affect the dependent variables. The factors considered in the adjusted models were age, sex, race, ejection fraction, and comorbidities such as high blood pressure, diabetes, and obesity. Statistical significance was determined at the level of 0.05.

## Results

### Demographic characteristics

A total of 1569 subjects were included in this study. The demographic characteristics of the subjects are presented in Table 1. The majority of samples were males (87.00%). Age was classified into early adult (20–39 years old), middle adult (40–59 years old), late adult (60–65 years old), and old age (> 65 years old). The majority of the subject classified as middle adults was 875 (55.77%), followed by late adults at 358 (22.82%), old age at 316 (20.14%), and the last early adult at only 20 (1.27%). A total of 385 patients (24.54%) had diabetes. Total ONCABG procedures were 1278 (81.45%), and OPCABG was 291 (18.55%). Intensive care unit length of stay (ICU LOS) was classified into short (≤ 2 days) and prolonged (> 2 days). Short ICU LOS was observed in 1115 cases (71.06%), and only 454 cases (28.94%) had prolonged ICU LOS. Mortality among CABG patients was 44 (2.8%) from the 1569 data collected. Among the 44 patients who died, nine were in the OPCABG group, and 35 were in the ONCABG group (Table 2).

**Table 1:** Study sample characteristics

Characteristics	Frequency (n = 1569)	Percentage (%)
*Sex*		
Male	1365	87.00
Female	204	13.00
*Age*		
Early adult, 20–39	20	1.27
Middle adult, 40–59	875	55.77
Late adult, 60–65	358	22.82
Old age, > 65	316	20.14
*Ejection fraction*		
Normal, > 50%	879	56.02
Mild, 40–50%	293	18.67
Moderate, 30–39%	230	14.66
Severe, < 30%	167	10.65
*CABG technique*		
On-pump	1278	81.45
Off-pump	291	18.55
*Blood pressure*		
Normotensive	989	63.03
Hypertensive	580	36.97
*Obesity*		
Non-obese	1390	88.59
Obese	179	11.41
*Diabetes mellitus*		
Non-diabetic	1184	75.46
Diabetic	385	24.54
*Length of stay*		
≤ 2 Days	1115	71.06
> 2 Days	454	28.94
*Mortality*		
Survived	1525	97.20
Died	44	2.80

**Table 2:** Intensive care unit and mortality comparison between groups

	OPCABG (*n* = 291)	ONCABG (*n* = 1278)
ICU LOS (days), mean ± SD	2.151 ± 0.100	1.572 ± 0.246
Mortality, *n* (%)	9 (3.09%)	35 (2.74%)

*ICU LOS* intensive care unit length of stay. *OPCABG* off-pump coronary artery bypass graft. *OCABG* on-pump coronary artery bypass graft. *SD* standard deviation

### Analysis of ICU length of stay and mortality

We found a significantly longer ICU LOS in OPCABG patients (2.151 ± 0.100 days) compared to ONCABG patients (1.573 ± 0.246 days) with a *p*-value of 0.028. Similar results were found when the models were adjusted for the effects of covariates, with an adjusted mean of ICU LOS of 3.146 (SD = 0.281) for OPCABG patients and 2.548 (SD = 0.245) for ONCABG patients. Analysis results of the CABG technique and ICU LOS are presented in Table 3. The analysis of the CABG technique and mortality by using logistic regression models shows that there was no significant difference in mortality between OPCABG and ONCABG patients both in the unadjusted model (OR 1.133, CI 95% 0.485–2.8) and the adjusted model (OR 1.133, CI 95% 0.482–2.817). Analysis results of the CABG technique and mortality are presented in Table 4.

**Table 3:** Univariate and multivariate analyses of CABG technique and ICU LOS

CABG technique	Intensive care unit length of stay					
	Univariate			Multivariate		
	Mean	SD	*P*	Mean	SD	*P*
OPCABG	2.151	0.100	0.028*	3.146	0.281	0.022*
ONCABG	1.573	0.246		2.548	0.245	

*P p-value for statistical significance at the level 0.05. *ONCABG* on-pump coronary artery bypass graft. *OPCABG* off-pump coronary artery bypass graft. *SD* standard deviation

**Table 4:** Univariate and multivariate analyses of CABG technique and mortality based on the logistic regression model

	Mortality					
	Univariate			Multivariate		
	OR	CI 95%	*P*	OR	CI 95%	*P*
OPCABG vs. ONCABG	1.133	0.485–2.800	0.733	1.133	0.482–2.817	0.735

*ONCABG* on-pump coronary artery bypass graft. *OPCABG* off-pump coronary artery bypass graft. *OR* odd ratio. CI 95% confidence interval 95%

## Discussion

### Intensive care unit length of stay in OPCABG and ONCABG

Our study found that the length of stay in the intensive care unit was significantly longer for patients who underwent OPCABG than those who underwent ONCABG. This finding highlights a discrepancy between recently published theories and the practices observed in our study centre. We reviewed the literature to explain this phenomenon. We identified several problems associated with the CABG procedure regardless of the technique, including transient left ventricular dysfunction, capillary leak, warming from hypothermia, and emergence from anaesthesia [6].

Despite advancements in surgical procedures and myocardial protection measures, the prevalence of transient left ventricular systolic failure remained consistent over the years [6]. OPCABG was expected to mitigate myocardial damage by reducing the inflammatory response. However, it is important to note that it does not entirely eliminate inflammation caused by surgical trauma and anaesthetic drugs. The increased systemic vascular resistance observed after surgery is likely due to decreased ventricular function rather than the primary cause of decreased cardiac contractility. Inflammation-induced generation of oxygen-free radicals and release of proteolytic enzymes damage endothelial cells, leading to capillary leak syndrome. Hypothermia affects coagulation, increases systemic vascular resistance, and contributes to oxygen consumption and carbon dioxide generation.

The advantages of OPCABG over ONCABG, such as avoiding the inflammatory effects of cardiopulmonary bypass (CPB), minimizing transient left ventricular dysfunction, and reducing capillary leak, suggest that issues with the anaesthetic strategy may contribute to longer ICU treatment courses in OPCABG patients. Anaesthetic goals for OPCABG include safe induction and maintenance of anaesthesia, adequate hemodynamic stability throughout surgery, and early emergence and ambulation supported by postoperative analgesia [7]. The anaesthetic technique discussed by Hemmerling et al. emphasizes maintaining heart rate, mean arterial pressure, and SvO2 within specific ranges, ensuring adequate preload and using vasopressors or inotropes to treat hypotension [8]. Fast extubation can be challenging for OPCABG patients due to hemodynamic instability caused by ischemia prevention and heart position during the operation. Hypotension during surgery may require increased fluid administration and vasoconstrictor agents, potentially leading to volume overload and delayed extubation [9].

While many studies have reported that OPCABG shortens hospital and ICU LOS [10–13], our findings contradicted this trend. The reasons behind the discrepancy in LOS were not discussed in previous articles. Our study suggests that the success of OPCABG in shortening LOS depends on various factors, including clinicians' preparedness, operator expertise, and the collaboration of supporting clinicians, such as anaesthesiologists and cardiologists, to achieve the desired outcomes. The aforementioned post-CABG problems and the consideration for extubation in OPCABG patients may explain the prolonged ICU LOS observed in our study, despite reports of shorter LOS in other centres. Further data from our centre are needed to provide a clearer understanding of this explanation. However, our study did not provide complete data on the mentioned problems, nor the data on hypotension, volume overload, vasopressor agent use, duration of intubation, and clinician's expertise due to the large amount of data that are not recorded completely. The specific factors influencing LOS in OPCABG require further investigation to improve outcomes and optimize patient care.

### Mortality in OPCABG and ONCABG

Factors such as patient tolerance, disease progression, procedural complexity, and postoperative recovery influence mortality rates in CABG surgery [14]. Complications are more common with ONCABG, which may explain its lower death rate than OPCABG [15]. However, our study found no significant difference in mortality between the two techniques.

ONCABG is associated with perioperative complications due to using a CPB machine and manipulating the ascending aorta, including myonecrosis, neurological deficits, renal dysfunction, and systemic inflammation. Strategies to address these issues have been explored, such as measuring brain injury using S100 beta serum concentrations and reducing neurocognitive dysfunction. Lipid material and particle matter have been highlighted as possible causes of postoperative neurocognitive dysfunction in blood collected from the operating field after on-pump CABG [16].

A systemic inflammatory response (SIRS) occurs after on-pump surgery due to surgical trauma, nonphysiological surfaces, ischemia-reperfusion, and hypothermia. Surgical trauma, contact of blood with nonphysiological surfaces (e.g. pump tubing, oxygenator surfaces), myocardial ischemia and reperfusion, and hypothermia all combine to cause a dramatic release of cytokines (e.g. interleukin (IL-6 and IL-8) and other inflammatory mediators after on-pump cardiac surgery. SIRS has been observed in patients undergoing CPB, prompting the development of measures to avoid or reduce its recurrence [17]. Various interventions have been attempted to prevent or reduce SIRS, but their impact on outcomes is unclear [16].

OPCABG, unlike ONCABG, performed on a beating heart with stabilizing devices, aims to minimize complications by avoiding CPB. By avoiding cardiopulmonary bypass, which is linked with microemboli formation, increased blood–brain barrier permeability, and aortic manipulation during cross-clamping and cannulation, OPCABG could theoretically reduce morbidity, notably stroke, and mortality. According to Head et al., the off-pump method was superior to ONCABG in terms of mortality [14]. Another study by Khan et al. [10] mentions that the risk of mortality increases with the ONCABG technique as it increases SIRS incidence, which can cause mortality from septic shock. However, Gaudino et al. report the contrary as they found that OPCABG is associated with a higher incidence of incomplete revascularization, an increased need for repeated revascularization, and decreased midterm survival compared with ONCABG.

On the other hand, our study concludes no significant difference in mortality between OPCABG and ONCABG. A study conducted by Brewer *et al.* [11] supports our findings as they demonstrate no difference in operative mortality for OPCAB patients compared to ONCAB patients. Hillis *et al*. [16] mention in the ACCF/AHA Guideline for Coronary Artery Bypass Graft Surgery that around the year 2005, an AHA scientific statement comparing the two techniques concluded that regardless of some studies about the comparison of both procedures, both generally resulted in excellent outcomes and that neither method should be considered superior to the other. Because CPB maintains systemic circulation, surgeons often favour ONCABG in patients with hemodynamic impairment. OPCABG, on the other hand, is favoured by some surgeons who have substantial experience with it and are, therefore, familiar with its technical aspects. According to the explanations, ONCABG was better for mortality than OPCABG because of the impacts of the CPB machine, SIRS, and cerebrovascular accidents. However, our study suggests no significant difference in mortality in both techniques in the authors' centre.

The choice between techniques depends on surgeon preference and patient characteristics. Our study has limitations, being retrospective and single-centre. Future research should include prospective studies or randomized controlled trials with long-term survival data to eliminate confounding factors further. Furthermore, our study was a single-centre study; thus, the applicability of the results was worth discussing. Additionally, the lack of data, such as risk stratification and the use of inotropes, has prevented us from allocating the sample into sub-groups. Further research with these data is needed to better understand our findings.

## Conclusions

Our study found that ICU LOS was significantly longer in OPCABG patients than in ONCABG patients, and there was no significant difference in mortality between the two groups. The results suggest that clinicians have to pay more attention to some suspected problems as the reasons behind OPCABG's prolonged ICU so that the goal of OPCABG application can be achieved. In addition, some improvements still need to significantly decrease mortality in applying the OPCABG procedure in the authors' centre.

## Data Availability

Data are not available for third-party use as the ethics approval does not cover this for this project.

## Abbreviations

CABG: Coronary artery bypass graft
CAD: Coronary artery disease
CVD: Cardiovascular disease
CPB: Cardiopulmonary bypass
ICU LOS: Intensive care unit length of stay
ONCABG: On-pump coronary artery bypass graft
OPCABG: Off-pump coronary artery bypass graft surgery
SIRS: Systemic inflammatory response
